# Transitioning Into Retirement and the Meaning of Home: A Qualitative Study Among the Younger Old in Sweden

**DOI:** 10.1093/geroni/igaa057.2003

**Published:** 2020-12-16

**Authors:** Maya Kylén, Charlotte Löfqvist, Maria Haak, Susanne Iwarsson

**Affiliations:** 1 CASE Lund University, Lund, Skane Lan, Sweden; 2 Lund University, Lund, Skane Lan, Sweden

## Abstract

Housing is the main spatial context for aging, important for well-being, a sense of identity and independence in daily life. Yet, as people grow older housing needs change and knowledge about how people reason about their future home when they enter retirement age is lacking. This qualitative study presents findings that explored meaning of home and health dynamics in the present and in a projected future among community-living people aged 67 – 70 years. Findings suggest that the home becomes progressively important after retirement. Not only the immediate home environment but also local neighborhoods influence perceptions about home. Home brings emotional and social benefits but also worries about how to cope with complex home ambivalence when reflecting upon future housing arrangements. The findings highlight the importance of considering perceived aspects of home and could be used to raise awareness among policymakers, housing authorities and professionals involved in housing-related counselling.

